# Adherence to “dietary approaches to stop hypertension” eating plan in relation to gastric cancer

**DOI:** 10.1186/s12937-020-00560-w

**Published:** 2020-05-11

**Authors:** Fatemeh Toorang, Bahareh Sasanfar, Maryam Hadji, Ahmad Esmaillzadeh, Kazem Zendehdel

**Affiliations:** 1 Department of Community Nutrition, School of Nutritional Sciences and Dietetics, Tehran University of Medical Sciences, Tehran, Iran; 2 Cancer Research Center, Cancer Institute of Iran, Tehran University of Medical Sciences, I, Tehran, R Iran; 3 Faculty of social science, Tampere University, Tampere, Finland; 4 Obesity and Eating Habits Research Center, Endocrinology and Metabolism Molecular-Cellular Sciences Institute, Tehran University of Medical Sciences, Tehran, Iran; 5 Department of Community Nutrition, School of Nutrition and Food Science, Isfahan University of Medical Sciences, Isfahan, Iran; 6 Cancer Biology Research Center, Cancer Institute of Iran, Tehran University of Medical Sciences, P.O. Box: 13145158, Tehran, I.R Iran; 7 Breast Diseases Research Center, Cancer Institute of Iran, Tehran University of Medical Sciences, I, Tehran, R Iran

**Keywords:** Gastric cancer, Dietary approach to stop hypertension, Diet, Case-control

## Abstract

**Background:**

Although adherence to “Dietary Approaches to Stop Hypertension” (DASH) dietary pattern has been linked with reduced risk of several cancers. To our knowledge no studies have examined the association between the DASH dietary pattern and risk of gastric cancer. This study was performed to investigate the association between adherence to the DASH dietary pattern and odds of gastric cancer in Iran.

**Methods:**

This hospital-based case-control study was conducted on 178 histo-pathologically confirmed patients with gastric cancer and 276 sex-matched healthy controls. A validated 146-item Diet History Questionnaire (DHQ) was used to assess participants’ usual dietary intakes. The DASH dietary pattern scores were calculated using the method introduced by Fung. Unconditional logistic regression, in which potential confounders were taken into account, was applied to determine the association of adherence to the DASH dietary pattern and odds of gastric cancer.

**Results:**

Mean age of cases and controls were 60.8 and 53.2 y, respectively. After controlling for age, sex and energy intakes, participants in the highest tertile were 62% less likely to have gastric cancer than those in the lowest tertile (OR 0.38; 95% CI 0.22,0.65; P_trend_ < 0.004). Further adjustment for other potential confounders, including education, marital status, residential place, alcohol intake and smoking, did not change the association dramatically (OR 0.44; 95% CI 0.25, 0.78; P_trend_ = 0.005). Even after additional controlling for H-Pylori infection and BMI, greatest adherence to the DASH dietary pattern was associated with a 54% decreased risk of gastric cancer (OR 0.46; 95% CI 0.26, 0.83; P_trend_ = 0.01).

**Conclusions:**

Adherence to the DASH dietary pattern was associated with lower gastric cancer risk in this case-control study.

## Background

Gastric cancer is one of the most common cancers in the world. Although its incidence is decreasing in most developed countries, it is still known as the forth common cancer in men and the seventh common cancer in women worldwide [1]. In Iran, the estimated Age-Standard Rate (ASR) is 26.1 per 100, 000 in men and 11.1 per 100, 000 in women [2]. As approximately 80% of patients are diagnosed in advanced stages and do not benefit from therapeutic strategies, primary prevention and screening for early detection are the most effective strategies in gastric cancer control [3].

Prevention strategies need to be designed based on established risk factors. Along with genetics, H.pylori infection, tobacco smoking, alcohol drinking and body fatness; diet has also been shown to play a major role [3–5]. For instance, there is strong evidence that consumption of salt-preserved foods or processed meats is positively associated with the risk [6–10]. A systematic review of 42 studies concluded that higher consumption of red meat was associated with a 67% increase in risk of gastric cancer (OR 1.67 comparing highest to lowest intake; 95%CI 1.36, 2.05). Higher consumption of processed meat was associated with considerable higher risk in this study (OR 1.76 comparing highest to lowest intake; 95%CI 1.51,2.05) [8]. A review of cohort studies indicates that risk of gastric cancer could increase by 12% per 5 g/day increase of salt intake [11]. The latest review by The World Cancer Research Fund and American Institute for Cancer Research reported that there is limited suggestive evidence on the association between fruit intake and risk of stomach cancer. It also asserted that the evidence of associations between other dietary intakes such as vegetables, nuts and dairy and risk of stomach cancer is limited and inconsistence [10]. It has been assumed that inconclusive findings on the diet-disease associations were due to assessment of individual nutrients or foods. Studying dietary patterns would be more reasonable and informative due to considering the interaction among foods and nutrients [12, 13]. Dietary Approaches to Stop Hypertension (DASH) eating pattern was developed and initially recommended for controlling blood pressure [14]. Its protective role in other conditions including obesity [15], diabetes [16], metabolic syndrome [17, 18] and CVD [19] was then demonstrated. This dietary pattern consists of high intake of fruit, vegetables, legumes, nuts, whole grains and low fat dairy, and low intakes of sodium, red and processed meats and sweetened beverages [20]. This dietary pattern is largely similar to the recommendations of American Cancer Society and International Agency for Research on Cancer (IARC) for cancer prevention [21, 22]. Many studies have investigated the association of the DASH dietary pattern components with several cancers [23]. Earlier publications have shown inverse association between adherence to the DASH dietary pattern and risk of colorectal [24] and breast cancers [25], but the association with gastric cancer has not been investigated so far. Moreover, studies on diet-disease associations, including cancer, mostly came from western countries and few data have been reported from the understudied region of Middle East [26–28]. Dietary cultures and environmental factors affecting the risk of cancer are discrepant in different areas. Traditional diet of Middle Eastern population included large amounts of refined grains and carbohydrates along with high intakes of salt and low intake of animal products [29, 30]. However, nutrition transition in these countries is occurring at an alarming rate and their dietary intakes are changing rapidly [29, 30]. Energy, total fat and animal proteins intakes are increasing in these region and intake of fruits, vegetables and dairy is decreasing at the same time [31–33]. Moreover, their gastrointestinal microbiota is different due to different dietary habit and life-style which could effect on association of dietary pattern and cancer risks [34]. Assessment of adherence to the DASH dietary pattern with gastric cancer is particularly relevant for Middle Eastern population due to their specific dietary patterns. This study was, therefore, done to explore the relationship between adherence to the DASH dietary pattern and risk of gastric cancer in Iran.

## Methods

### Participants

This hospital-based case-control study was conducted in the Cancer Research Center, Imam Khomeini complex, Tehran University of Medical Sciences, Tehran, Iran between May 2010 and June2012. Histo-pathologically confirmed patients with gastric cancer were recruited in the study. Patients were referred to this hospital from all parts of Iran. The inclusion criteria for case enrollment were diagnosed with gastric cancer within previous 6 months with no medical history of any cancer and age of 40 years or older. Convenience sampling method was used for case enrollment. In total, 276 controls were recruited from the same hospital based on non-random sampling method if they were apparently healthy persons. They were chosen among those who came to visit their relatives in the hospital. The response rate was 95% among cases and 70% among controls.

### Ethics

Written informed consent was provided by all participants after face to face description of study protocol and aims. Ethical committee of Cancer Research Center, Tehran University of Medical Sciences reviewed and approved the study protocol.

### Assessment of dietary intakes

We used a validated 146-item Diet History Questionnaire (DHQ) to assess participants’ dietary intakes. A detailed description of DHQ, its development and validity has been explained in details elsewhere [35]. Briefly, it included 146 questions related to the past 12-month consumption of foods and Iranian mixed dishes. Trained nutritionists conducted face to face interview to complete the (DHQ). The interviewers asked participants to recall their dietary intakes based on a given portion size in the preceding year. Patients with gastric cancer were requested to recall their intakes before the appearance of cancer symptoms. Participants were able to choose their frequency consumption of different foods and dishes based on Iranian home scales such as spoon, plate, bowl, ladle or splatter. DHQ data were converted to grams/day using the booklet of household measures [36]. Then daily intakes of energy and all micro and macro-nutrients were computed using translated version of McCance and Widdowson’s Food composition table modified for Iranian foods [37–39]. We also asked participants about their supplement use during the previous year.

Previous validation study of DHQ in this population revealed good correlation coefficients between nutrients assessed by DHQ and multiple 24-h recalls completed over a year [35]. Deattenuated spearman correlation coefficients of equal or greater than 0.5 were obtained for energy, carbohydrate, protein, fiber, vitamin A, carotene, niacin, folate, vitamin B12, biotin, vitamin C, sodium, magnesium, iron, zinc, selenium between DHQ and the average of 24-h dietary recalls [40].

### Construction of DASH score

The DASH dietary pattern scores were calculated using the method introduced by Fung [41]. DASH diet score were constructed based on nutrients and foods minimized or emphasized in the DASH dietary pattern. It emphasizes high intake of fruit, vegetable, nuts, legumes, low fat dairy products, and whole grains and recommends low intake of sodium, sweetened beverages and red and processed meats [20, 41]. As the Iranian population mainly consumes refined grains [42], therefore, lower intake of grains was considered as a protective factor. Initially, we obtained energy-adjusted amount of components of the DASH dietary pattern using residual method [43]. We classified participants into quintiles according to intakes of each components of the DASH dietary pattern. Quintile cut-off points of these components were obtained based on intakes in control subjects in order to avoid probable bias that might be arise due to the changes in dietary intakes in patients. Then, in terms of fruit, vegetable, nuts, legumes and low fat dairy products participants were given the score of 1 if they placed at quintile 1. This was done for all quintile of these components such that those in the top quintile of these food items were given the score of 5. We did vice versa for sodium, sweetened beverages and red and processed meats; such that those in the bottom quintile of these food items were given the score of 5 and those in the top quintile were given the score of 1. The overall DASH score for each participant was calculated by summing up all components’ scores. The total score ranged from 8 to 40 [41, 44]. The greater the score presents the great adherence to the DASH dietary pattern.

### Assessment of gastric cancer

Diagnosis of gastric cancer was done based on gastroscopic or surgical biopsy reviewed by an experienced pathologist. Patients with histologically confirmed stomach cancer as defined by the second edition of the International Classification of Diseases for Oncology (ICDO-c16) were enrolled**.** We only recruited patients who had been diagnosed within the 1 yr prior to the date of interview.

### Assessment of covariates

Demographic and general information were collected using a structured questionnaire through a face to face interview conducted by a trained bachelor of health sciences. These included information about gender, marital status, education, residential places and smoking habits. Data on usual weight and height were collected through self-reported method. We did not examine current weight due to the effect of gastric cancer on weight in these patients. Body Mass Index (BMI) was calculated as weight in kilograms divided by height in meters squared. Smoking status was examined through asking participants about their usual behavior on smoking during the last year. They were classified as current smokers and non-smokers. To examine *H. pylori* infection, we took 10 ml of venous blood samples from all participants at fasting or non-fasting state when they attended the center. Serum samples were evaluated for IgG antibody using ELISA kits. Experienced technicians, who were not aware of the study design and case/control status of donors, performed the serologic assays. The H.pylori antibody test was repeated in a random selection of serums to ascertain validity. The seropositivity was defined as the presence of antibody and seropositivity > 0.87 was considered as positive.

### Statistical analysis

Characteristic of patients with gastric cancer and controls were compared using Student’s independent t test for continuous variables and chi-square test for categorical variables. Comparison of these variables across tertiles of DASH diet score was done using chi-square test for categorical and one-way ANOVA for continuous variables. To determine the association of adherence to the DASH dietary pattern and odds of gastric cancer, we applied unconditional logistic regression models, in which several potential confounding variables were taken into account. In these analyses, first we controlled for age (continuous), sex (male, female) and energy intake (continuous). Then, further adjustments were done for education (categorical), marital status (single, married) and residential place (Tehran, other cities). Alcohol intake (g/day) and smoking status (ever vs. never) were taken into account in an additional model and finally, we controlled for H-pylori infection (positive, negative). To identify independent-of-obesity association between adherence to the DASH dietary pattern and gastric cancer, we also adjusted for BMI (continuous). In all these analyses, the first tertile of DASH diet score was considered as a reference and the odds ratios (ORs) and 95% CIs for gastric cancer were calculated. The trend of odds ratios was examined by considering the median score of the DASH dietary pattern in each tertile as a continuous variable. All statistical analyses were carried out using STATA (STATA, version 14, State Corp., College station, TX).

## Results

Study participants were 178 patients with gastric cancer and 276 healthy controls. Table 1 shows the distribution of patients and controls according to selected covariates. Characteristic of participants across tertiles of DASH diet score are also provided in this table. Patients with gastric cancer were older (60.8 vs. 53.2 y, *P* < 0.001) and more likely to be males (74.2 vs. 63.8%, *P* = 0.02), married (97.8 vs. 85.9%, P < 0.001) and illiterate (62.4% vs. 26.1%, P < 0.001) than controls. They were less affected with *H. pylori* (38.2 vs. 56.16%, P < 0.001) and were less likely to be current smokers (54.5% vs. 62.2%, *P* = 0.01) than controls. When examined across tertiles of DASH diet score, we found that greater adherence to the DASH dietary pattern was not associated with any difference in covariates except for marital status (P = 0.01).

**Table 1:** General characteristic of participants across tertiles of DASH score^1^

Characteristics	Groups			Tertile of DASH score			
	Cases (*n* = 178)	Controls (*n* = 276)	P^2^	1 (*n* = 176)	2 (*n* = 158)	3 (*n* = 120)	P^3^
Age (years)	60.8 ± 12.0	53.2 ± 11.9	< 0.001	54.5 ± 12.9	57.6 ± 12.7	56.6 ± 11.4	0.07
Alcohol intake (g/day)	6.8 ± 86.4	1.7 ± 11.7	0.3	1.4 ± 9.9	8.4 ± 92.4	1.11 ± 9.6	0.44
BMI (kg/m2)	27.8 + 16.4	26.0 + 8.2	0.12	27.6 ± 14.2	26.4 ± 13.3	25.8 ± 4.6	0.44
Gender (Male, %)	132(74.2)	176(63.8)	0.02	108(65.9)	122(71.4)	78(65.6)	0.46
Marital status (Married, %)	174(97.8)	237(85.9)	< 0.001	156(95.1)	157(91.8)	100(84.0)	0.01
Education (illiterate, %)	111(62.4)	72(26.1)	< 0.001	69(42.1)	68(39.8)	46(38.7)	0.53
Residential (Tehran, %)	93(52.3)	140(50.8)	< 0.001	86(52.4)	92(53.8)	55(46.1)	0.42
Smoking (yes %)	81(45.5)	85(30.8)	0.001	62(37.8)	66(38.6)	38(31.9)	0.47
H.pylori (positive, %)	68(38.2)	155(56.2)	< 0.001	82(50.0)	83(48.5)	58(48.7)	0.96

^1^ Reported figures are means±SDs unless indicated

^2^Obtained from chi-square test for categorical variables and independent student’s t-test for continuous variables

^3^ Obtained from chi-square test for categorical and one-way ANOVA for continuous variables

Dietary intakes of participants are shown in Table 2. Compared with controls, patients with gastric cancer had significantly lower intakes of grains (313 vs. 443, *P* < 0.001), vegetables (242 vs. 378, P < 0.001) and fruits (372 vs. 554, P < 0.001). As expected, greater adherence to the DASH dietary pattern was associated with higher intakes of nuts and legumes (P < 0.001), vegetables (P < 0.001), fruits (P < 0.001) and low fat dairy (P < 0.001). Subjects with greater adherence to the DASH dietary pattern had lower intakes of grains (*P* = 0.01), sweetened drinks (*p* = 0.001), and red and processed meats (*p* = 0.002).

**Table 2:** Dietary intakes of participants across tertiles of DASH score

Nutrient /food group	Groups			Tertiles of DASH diet score			
	Cases (*n* = 178)	Controls (*n* = 276)	P^1^	1 (*n* = 176)	2 (*n* = 158)	3 (*n* = 120)	P^2^
Energy (Kcal/d)	2853 ± 1241	2782 ± 1252	0.55	2678 ± 1154	2871 ± 1325	2903 ± 1247	0.23
Grains (g/d)	313 ± 272	443 ± 218	< 0.001	440 ± 282	388 ± 291	332 ± 268	0.01
Nuts and legumes (g/d)	37 ± 28	43 ± 34	0.09	34 ± 28	39 ± 29	51 ± 38	< 0.001
Vegetables (g/d)	242 ± 172	378 ± 221	< 0.001	249 ± 189	310 ± 193	448 ± 220	< 0.001
Fruits (g/d)	372 ± 278	554 ± 392	< 0.001	365 ± 268	449 ± 307	694 ± 451	< 0.001
Low fat dairy (g/d)	588 ± 580	582 ± 65	0.09	275 ± 433	651 ± 615	914 ± 681	< 0.001
Red and processed meats (g/d)	31 ± 29	34 ± 36	0.27	40 ± 39	31 ± 29	27 ± 28	0.002
Sweetened drinks (g/d)	85 ± 89	82 ± 119	0.08	105 ± 91	79 ± 95	59 ± 139	0.001
Fats (g/d)	105 ± 58	98 ± 61	0.23	92 ± 49	102 ± 61	109 ± 69	0.06
Proteins (g/d)	126 ± 51	123 ± 47	0.49	125 ± 51	123 ± 49	126 ± 46	0.83
Carbohydrates (g/d)	367 ± 181	365 ± 177	0.90	351 ± 166	381 ± 200	365 ± 159	0.32
Potassium (mg/d)	5682 ± 2478	5539 ± 2245	0.52	5497 ± 2293	5620 ± 2413	5694 ± 2305	0.77
Sodium (mg/d)	2686 ± 1727	2617 ± 1477	0.65	2818 ± 1742	2669 ± 1678	2367 ± 1093	0.06
Calcium (mg/d)	2253 ± 1285	2218 ± 1193	0.77	2223 ± 1240	2227 ± 1209	2249 ± 1251	0.98
Folate (mg/d)	424 ± 178	414 ± 164	0.53	414 ± 164	418 ± 175	421 ± 169	0.93

^1^using independent student T-test

^2^ using one-way ANOVA

Multivariable adjusted ORs for gastric cancer across tertiles of DASH diet score are provided in Table 3. Before adjusting for covariates, adherence to the DASH dietary pattern was inversely associated with gastric cancer (OR for comparing extreme tertiles: 0.47; 95% CI 0.28–0.77; P_trend_ = 0.004). After controlling for age, sex and energy intakes, greater adherence to the DASH dietary pattern was associated with a substantial reduced odds of gastric cancer; such that participants in the highest tertile were 62% less likely to have gastric cancer than those in the lowest tertile (OR 0.38; 95% CI 0.22,0.65; P_trend_ < 0.001). Further adjustment for other potential confounders, including education, marital status, residential place, alcohol intake and smoking, did not change the association dramatically (OR 0.44; 95% CI 0.25, 0.78; P_trend_ = 0.005). Even after additional controlling for H-Pylori infection and BMI, greatest adherence to the DASH dietary pattern was associated with a 54% decreased risk of gastric cancer (OR 0.46; 95% CI 0.26, 0.83; P_trend_ = 0.01).

**Table 3:** Odd Ratios (ORs) and 95% confidence Intervals (CIs) for gastric cancer across tertiles of DASH score^a^

	OR (95%CI)			P trend^a^
	Tertile 1	Tertile 2	Tertile 3	
Total No. of cases/controls (178/276)				
Crude	1.00	0.86(0.56–1.33)	0.47(0.28–0.77)	0.004
Model A^b^	1.00	0.68(0.43–1.09)	0.38(0.22–0.65)	< 0.001
Model B^c^	1.00	0.73(0.44–1.19)	0.43(0.24–0.76)	0.004
Model C^d^	1.00	0.73(0.44–1.21)	0.44(0.25–0.78)	0.005
Model D^e^	1.00	0.73(0.44–1.22)	0.43(0.24–0.77)	0.005
Model E^f^	1.00	0.74(0.44–1.25)	0.46(0.26–0.83)	0.01

^a^ Trend based on median values of each tertile

^b^Adjusted for age (continuous), sex (male/female) and energy intake (continuous)

^c^Further adjusted for education (illiterate/literate), marital status (married/single) and residential place (Thran/others)

^d^ Additionally adjusted for alcohol intake (continuous) and smoking status (smoker/nonsmoker)

^e^ Further adjusted for H.pylori infection (positive/negative)

^f^ Additionally controlled for BMI (continuous)

## Discusion

In this large hospital-based case control study, we found an inverse association between adherence to the DASH dietary pattern and odds of gastric cancer. To the best of our knowledge, this study is the first examining the association between adherence to the DASH dietary pattern and risk of gastric cancer.

As mentioned before, the DASH dietary pattern was initially suggested to manage hypertension [20]; however, its beneficial effects on other health outcomes including some cancers have also been reported [45–49]. Although no prior information are available about the link between this dietary pattern as a whole and gastric cancer, several studies reported significant associations between components of the DASH dietary pattern and risk of gastric cancer [50]. For instance, high intake of fruits and vegetables, as main components of this dietary pattern, was inversely associated with gastric cancer [51]. However, the comprehensive report on nutrition and cancer prevention by the World Cancer Research Fund and American Institute for Cancer Research revealed that the evidence on the association of fruit and vegetable intake and risk of gastric cancer is not convincing. Based on this report, there is limited evidence to suggest that fruit consumption is protective against gastric cancer. Other dietary components were reported as limited evidence, no conclusion [10].

Several studies have examined the association between other healthy dietary patterns and risk of gastric cancer [26, 52, 53]. In a review by Schwingshackl and Hoffmann, consumption of Mediterranean-type diet was associated with a 27% reduced risk of gastric cancer. There was almost a 2-fold difference in risk of gastric cancer, comparing adherence to a healthy diet rich in fruit and vegetables and a western/unhealthy diet rich in starchy foods, meat and fats [54]. However, some studies did not suggest any significant association. Analyzing data from National Institutes of Health-AARP Diet and Health Study, the investigators found no significant association between adherence to healthy diet, as measured by Healthy Eating Index, or Mediterranean diet and risk of gastric cancer [26]. Therefore, it seems that there are unanswered questions about the association between dash or other dietary patterns and risk of gastric cancer; hence further studies are required to shed light on this issue in the future.

Patients with gastric cancer in this study were less likely to be affected by H-Pylori infection and reflux. This is in opposite to earlier publications [55, 56]. It should be kept in mind that we evaluated *H. pylori* infection by assessing Ig G antibody which might be cleaned during the gastric atrophy and tumor growth [57]. It is recognized that, H.pylori did not colonize in areas affected by cancer, metaplasia or atrophy and it is lost through development of advanced gastric diseases. This might explain why the patients with gastric cancer had H-pylori seronegative in the current study. Moreover, patients with gastric cancer were more likely to receive anti H.pylori treatments which can further explain this finding [58].

The mechanisms through which adherence to the DASH dietary pattern affect risk of gastric cancer are unknown. Prior investigations have shown the involvement of oxidative stress in the pathophysiology of several cancers [59–61]. The DASH dietary pattern contains high amounts of fruits, vegetables, whole grains, legumes and nuts. These foods are rich sources of dietary fiber, phenolic compounds, folate and carotenes; the beneficial effects of them on oxidative stress has earlier been shown [62, 63]. On the other hand, low consumption of red and processed meats and sweetened drinks might further help explaining the protective association between this dietary pattern and gastric cancer [64]. Red and processed meats contain high amounts of N-Nitroso compounds (NOCs), heterocyclic amines and polycyclic aromatic hydrocarbons [8, 65]. In addition, red meats are rich in iron and saturated fatty acids which have been shown as carcinogenic factors in the literature. Sweetened beverages are high in fructose; greater intake of which is a risk factor for gastric cancer. In addition, the DASH dietary pattern, probably through lower intakes of simple carbohydrates and higher intakes of fiber, is inversely associated with insulin resistance [66], which in turn is positively associated with risk of several cancers including gastric cancer [67]. Insulin and insulin-like growth factor 1 can promote cancer development by activating several signaling pathways associated with an elevated risk of oncogenesis [65].

### Strengths and limitations

High rates of participation, the same socioeconomic status of patients and controls, measuring the seropositivity of *H. pylori* as a risk factor for gastric cancer and the use of a validated FFQ for dietary assessment are strengths of this study. However, several limitations should also be noted. As with all epidemiological studies applying FFQ, misclassification of study participants based on their dietary intakes is unavoidable [68]. We used energy adjusted intakes of food groups to compute adherence to the DASH dietary pattern [43]. This can help reducing the possibility of misclassification. All food groups in the DASH dietary pattern were given an equal weight, while some foods might have greater effects than others in gastric cancer development. Although we controlled for several confounders, the possibility of residual confounding cannot be excluded. Other limitations of this study include a relatively small number of cases which did not allow meaningful analysis by separate histological type or tumor site. Given the case-control design of the study, the inherent limitations of recall and selection bias should also be considered [69].

## Conclusion

One the basis of this case-control study, we demonstrated that adherence to the DASH dietary pattern was inversely associated with risk of gastric cancer. This finding supports the current recommendations by several international guidelines to consume high amounts of plant based foods in the usual diet. It is clear that, further studies, in particular of prospective design, are required to confirm these findings. However, as the benefits of this dietary pattern have been firmly confirmed in several health conditions, it would be sensible to advice DASH dietary pattern to enhance public health.

## Data Availability

The datasets used and/or analyzed during the current study are available from the corresponding author on reasonable request.
